# Who Am I: The Conscious and the Unconscious Self

**DOI:** 10.3389/fnhum.2017.00126

**Published:** 2017-03-17

**Authors:** Michael Schaefer, Georg Northoff

**Affiliations:** ^1^Fakultät Naturwissenschaften, Medical School BerlinBerlin, Germany; ^2^Mind, Brain Imaging and Neuroethics, Institute of Mental Health Research, University of OttawaOttawa, ON, Canada

**Keywords:** self, cortical midline structures, neuroscience, embodiment, somatosensory cortex

## Abstract

Who am I? What is the self and where does it come from? This may be one of the oldest problems in philosophy. Beyond traditional philosophy, only very recently approaches from neuroscience (in particular imaging studies) have tried to address these questions, too. So what are neural substrates of our self? An increasing body of evidence has demonstrated that a set of structures labeled as cortical midline structures are fundamental components to generate a conscious self. Moreover, recent theories on embodied cognition propose that this conscious self might be supplemented by additional structures, for example, in the somatosensory cortices, which enable our brain to create an “embodied mind”. While the self based on cortical midline structures may be related to a conscious self, we here propose that the embodied facet of the self may be linked to something we call unconscious self. In this article we describe problems of this model of a conscious and unconscious self and discuss possible solutions from a theoretical point of view.

## Who Am I?

We know that even in prehistoric times humans tried to open the skulls of their sick conspecifics. Moreover, prehistoric men used human skulls, usually those of ancestors, for religious worship long after death. Thus, the head always seemed to be an object of interest for us. Perhaps the prehistoric men assumed that something inside our skull may be related to our feelings, thoughts and memories. But we had to wait until the French philosopher René Descartes, who was the first one who made the distinction between mind and body very explicit. His famous philosophical statement “Cogito ergo sum” can be translated as “I think, therefore I am”. Hence, he concludes that he can be certain that he exists because he thinks. For many researchers these thoughts mark the beginning of modern western philosophy. Descartes statement raised a lot of questions, in particular about the relationship between body and mind, which are still a matter of discussion today.

This is in particular true since modern neuroscience started to unravel the mystery of the brain. New imaging tools such as fMRI enable us to look at our brain while it is working. These new approaches have opened the door to answer the questions Descartes posed about the relationship between mind and body in a way he never would have imagined.

In this article we suggest the idea that the processing of self-referential stimuli in cortical midline structures may represent an important part of the conscious self, which may be supplemented by an unconscious part of the self that has been called an “embodied mind” (Varela et al., 1991), which relies on other brain structures.

## The Conscious Self: Cortical Midline Structures

Since the famous words by René Descartes there were numerous attempts to clarify what he described as a self. Descartes suggested that the self is a substance, such as a thing, which can be confronted with the body. But if so, how and where do these two substances interact? Remarkably, Descartes suggested a place where this interaction should take place: the epiphysis cerebri. Descartes believed this region to be the principal seat of the soul. In contrast, the Scottish philosopher David Hume argued that there is no self as a mental entity; there is only a complex set of perceptions of interrelated events that reflect the world. Hence, there are only events that we perceive. In this view, the self is merely an illusion. Similar, the contemporary German philosopher Thomas Metzinger argues that there is no self as a mental entity (Metzinger, 2003).

More recently, this problem has also been discussed in neuroscience. In order to examine the self in a neuroscientific way, studies focused on different features of the self. Central features of the self may include feelings of agency, ownership feelings for the body, autobiographical memory, experiencing the self as a unit, or labeling of stimuli as self-referential. Depending on the feature of the self that has been examined, the neuroscientific approach varied. For example, research on the last facet focused on self-referential relative to non-self-referential tasks. In a typical experimental paradigm Kelley et al. (2002) asked participants to judge trait adjectives (e.g., aggressive or friendly) as to whether they properly described themselves, a given case, or the current US president. Thereby, stimuli were categorized as self-relevant, other-referential, or case referential. Brain regions associated with self-relevant stimuli are then interpreted as describing the neural signature of our self (Kelley et al., 2002).

In spite of these different approaches, an increasing body of evidence consistently identified regions located in the midline of the human cerebral cortex, which have been labeled as cortical midlines structures (CMS), to be crucial for self-specific processing (Northoff, 2004, 2011, 2013, 2016). It has been suggested that those structures are central for self-relevant or self-related processing, thus enabling us to link internal with external stimuli (Northoff, 2016). Self-related processing describes the processing of a stimulus in relation to (but not representing it in) the self.

What are the structures of the CMS and how are they related to the self? The CMS structures include several phylogenetic old brain structures. They subserve different functions for establishing a self. For example, the orbitomedial prefrontal cortex (OMPFC) has been linked to a continuous representation of self-referential stimuli. The supragenual anterior cingulate cortex (SAC) seems to monitor these self-referential stimuli, while the dorsomedial prefrontal cortex (DMPFC) may evaluate them with respect to the relevance for the self. For example, the DMPFC and the SAC were involved when participants were asked to monitor and judge whether auditory verbal feedback was their own or another person’s voice (McGuire et al., 1996). The posterior-cingulate cortex (PC) may then be important to integrate these stimuli into the emotional and autobiographical framework of the person (Northoff and Bermpohl, 2004; Northoff, 2016).

The CMS can be understood as an anatomical unit because these regions maintain strong and reciprocal projections among each other. In addition, they demonstrate a similar pattern of connectivity to brain regions outside the CMS, e.g., to the ventro- and dorsolateral prefrontal cortex or the limbic system including hippocampus, amygdala and insula.

It is intriguing that this network of CMS overlaps with another network, the resting state or default mode network (DMN). This DMN describes interacting brain regions and is most commonly active when a person is not focused on the outside and the brain is at wakeful rest. The DMN is involved during passive rest, mind wandering, remembering the past and planning the future, and also when thinking about others. Among others, the DMN includes brain regions such as the medial prefrontal cortex, the angular gyrus and structures of the hippocampal formation (Huang et al., 2016).

So what kind of self do the CMS represent? Existing studies investigated the relationship between the CMS and the self predominantly with the focus on the ability to think about oneself. This is also supported by the link to the resting state activity network. Since one cannot think about oneself without being conscious, we here describe the CMS as representing in particular an important part of the conscious self. This conscious self represents a stable self over time, allowing us, for example, to travel through time (remembering the past and projecting into the future).

## The Unconscious Self: Embodiment

In the previous section we argued that a set of brain structures labeled as CMS is an important part of a conscious self. We here suggest that there are also unconscious parts of the self. The distinction between conscious and unconscious self is important because it points to the observation that our self is not limited to the stream-of-consciousness but includes also other features. These other features may include, for example, unconscious parts of the self. The concept between conscious and unconscious parts of the self is famous at least until the work of Freud. However, we here call processes as unconscious when thinking about the self usually does not tell us anything about these processes. In this sense, unconscious processes are automatic. We assume that there are numerous processes in our mind that can be described as unconscious. In this article we focus on a particular line of research, because studies based on this approach suggest convergent anatomical substrates underlying these unconscious facets of the self. Thus, we propose that embodied cognitions may represent important aspects of the unconscious self.

What is embodiment? There are different theories of embodiment and definitions. Embodiment in the most general form argues that human mental functions are shaped by the way the human body interacts with the world (Wilson, 2002). Mind, body and environment influence one another in order to promote adaptive success (Thompson and Varela, 2001; Wilson, 2002; Gallagher, 2005; Barsalou, 2008). In this sense, the body is an interface between the mind and the world, it merges our thoughts with the space around us (Varela et al., 1991). Gallagher points out that embodiment works preceding to any knowledge, it is not accessible to our consciousness. Therefore, Gallagher concludes that the body shapes the mind at a fundamental basic level, while it remains behind the scene (Gallagher, 2005).

What are neural substrates of this embodiment? Research on the conceptual or embodied metaphor theory (Lakoff and Johnson, 1999; Williams et al., 2009; Lakoff, 2014) provides suggestions about the neural underpinnings of embodied cognitions. Conceptual metaphors are different from linguistic metaphors. While linguistic metaphors are obviously present in language, conceptual metaphors mean understanding and experiencing one kind of thing in terms of another (Lakoff and Johnson, 1980). Numerous studies demonstrate how those embodied metaphors build a scaffold and guide our everyday behavior in an unconscious way (Lakoff and Johnson, 1999). An example is the moral purity metaphor, which binds moral purity and physical cleanliness (Zhong and Liljenquist, 2006). Studies on this metaphor have demonstrated that hand washing make us judge subsequent scenarios describing moral transgressions as less severe (Schnall et al., 2008). Hence, abstract thoughts about morality can be unconsciously grounded in sensory experiences. What are neural substrates related to these conceptual metaphor effects? Several studies determined primary motor and especially primary somatosensory cortices as crucial neural underpinnings of the embodied cognitions (Lacey et al., 2012; Schaefer et al., 2014). For example, it has been demonstrated that the moral-purity metaphor is related to sensory brain areas (Schaefer et al., 2015; Denke et al., 2016). This is also in line with recent theories on embodied simulation processes. Simulation here means that the retrieval of conceptual meaning involves a partial re-enactment of sensory and motor experiences (Gallese and Lakoff, 2005). The above-mentioned imaging studies provide support for this assumption.

But how can primary somatosensory areas be linked to embodied metaphors? In the traditional view those brain areas are known to represent primary modalities. Thus, the classic understanding of the primary somatosensory cortex is to reflect touch on the body surface in a more or less mechanical way (Kaas, 2008). However, recent findings in neuroscience draw the attention to more complex functions of the primary somatosensory cortex, pointing to a role for the somatosensory cortices in perceiving rather than reflecting touch on the body surface. Moreover, these brain areas seem to include even social perceptions such as empathy (Keysers et al., 2010; Schaefer et al., 2012). In his neural reuse theory, Anderson argues that brain areas may be involved in different neural partnerships depending on tasks and circumstances (Anderson, 2014). According to Anderson, “neural reuse” refers to a form of neuroplasticity, in which neural elements originally developed for one purpose are put to multiple uses. Embodied metaphors are examples of how our brain uses old strategies in new ways. Hence, higher-order cognitive processes such as moral thinking may be just recombinations of more simple and basic unconscious brain processes.

The brain areas representing the embodied self (in particular the sensorimotor brain areas) are different from the ones we have mentioned to be engaged in the CMS. We suggest that, while the CMS represent a conscious self, brain structures engaged in embodied cognitions might be related to an unconscious self. At least part of this unconscious self may be based on sensorimotor brain areas. We further assume that both parts of the self are consistently interacting.

But aren’t we often conscious about sensorimotor activation? And doesn’t this speak against a role of the sensorimotor brain areas for unconscious parts of the self? In fact, we are frequently aware of sensorimotor activation. However, often this activation is also automatic and unconscious. Again, we argue with Anderson that brain areas can have multiple roles. Based on the theory of embodied cognitions we assume that many conceptual metaphors (e.g., cleanliness and moral purity) were once learned consciously and now represent an unconscious link in our self (Lakoff and Johnson, 1980).

## How Does the Conscious Self Interact with the Body and the Environment?

The suggestion of a conscious self and an embodied self as an unconscious self that provides a link to our body experiences raises a number of problems. We will here discuss only one major point, which refers to the way the conscious self may be related to the embodied self. In contrast to Descartes’ suggestion, previous work has described this self as a brain based structure and organization, rather than a mental or physical entity located somewhere in the brain (Northoff, 2013). This conscious self as a structure or organization is related to both the body and the social world.

How can we imagine these relationships? When we describe the self as structure and organization we understand it as a system. But the concept of the embodied self states that the self or cognition is not an activity of the mind alone, but is distributed across the entire situation including mind, body, environment (e.g., Beer, 1995), thereby pointing to an embodied and situated self. How can a system include also its environment? According to the British philosopher and mathematician Georg Spencer-Brown a system is defined by its border, which not only separates the system from the environment, but also is the way a system is defined in the very first beginning: draw a distinction and a universe comes into being (Spencer-Brown, 1969).

Wilson suggests that the embodied self is an open system. Thus, the boundaries of a system are partly a matter of judgment and depend on the particular purposes of one’s analysis (Wilson, 2002). But we still need to ask what determines the border in those cases. Recent general system theory here provides an interesting view. Systems such as the consciousness have been described as functionally closed, which means they are systems that are separated from other systems and their environment by the specific way they operate (Luhmann, 1985, 1988, 1995). In this view, our consciousness is a closed system, which is built out of thoughts and nothing else. We can imagine this system as a self-referential system, in which every thought is followed by another thought, which is again followed by the next thought and so on. This is also called an “autopoietic” system (Luhmann, 1995). In this way, the self is a closed system, because both the situation as well as the body belong to the environment for this system (Luhmann, 1995). However, this system is only closed in the way it works, but it is open for incoming information from the social situation or from the body, e.g., responses from another individual or information that the body feels warmth. Interestingly, the self as an autopoietic system cannot be directly steered, it can only be perturbed. Thus, the self-referential circle is still closed, but can be “touched” or disturbed by information from the environment (e.g., feelings of warmth or friendly responses by a conspecific). The system itself then needs to make sense out of this “disturbance”, interpreting it in this or another way.

In this way, the conscious self may be both at the same time, open and closed. We further suggest that the unconscious self, which we described (at least partly) as the embodied self, represent one way the environment (e.g., the social world via the own body) may affect (disturb, perturbate) the conscious self. Therefore, given that at least parts of the unconscious self might be embodied, the mind also needs to be understood in the context of its relationship to a physical body that interacts with the world.

However, it remains unclear which neural structures carry this interaction of the conscious with the unconscious self. Future work is needed to supplement this conceptual relationship with neural substrates.

Furthermore, we argue that through embodiment the self is also embedded in the environment. This means that our self is not isolated but intrinsically social. The social dimension of the self has been discussed by numerous philosophers, often addressed as the question for intersubjectivity.

Hence, the self should not be understood as an entity located somewhere in the brain, isolated from both the body and the environment. In contrast, the self can be seen as a brain-based neurosocial structure and organization, always linked to the environment (or the social sphere) via embodiment and embeddedness. We further argue that embeddedness is first and embodiment may show up in a later developmental stage. The structure and organization that may define our self develops through childhood and adolescence. While the self is embedded in the environment from the very first beginning, embodiment may show up later in this progress. Furthermore, considering that there is no self without environment, we argue that the environment created the self.

Thus, we conclude that the self is part of a broader environmental system, including body and social dimensions. The brain’s cortical midline structures activity seem to be a neural predisposition for this constitution, which is at the same time dependent on the environmental context.

## Who We Are: The Conscious and the Unconscious Self

Who am I? Since human evolution once reached the state of an elaborated conscious self, we questioned ourselves these kinds of philosophical questions. And since at least the work of Sigmund Freud it is well known that the self includes also areas beyond our consciousness.

In this article we made the suggestion that the conscious self can be related to a network of brain areas that has been labeled as CMS. Moreover, we aimed to show that there are additional unconscious parts of the self; at least parts of them we here called embodied self, which may be based in particular on sensorimotor brain regions. Furthermore, we tried to describe the interaction between both systems by suggesting that the conscious self is a functionally closed (or autopoietic) system that can be disturbed by the unconscious embodied self. We are aware that these are very preliminary considerations. Furthermore, we again stress that the embodied self may represent only parts of the unconscious self. However, we believe that both future neuroscience unraveling as well as philosophical or theoretical advancements may further help us in the understanding of the self, one of the most peculiar achievements of the human evolution.

## Author Contributions

MS and GN wrote the manuscript.

## Conflict of Interest Statement

The authors declare that the research was conducted in the absence of any commercial or financial relationships that could be construed as a potential conflict of interest.
